# ASO Author Reflections: Disparities in Triple-Negative Breast Cancer Care: Are Some Patients Being Overlooked?

**DOI:** 10.1245/s10434-024-15640-y

**Published:** 2024-06-29

**Authors:** Pallavi Jonnalagadda, Saurabh Rahurkar

**Affiliations:** 1 The Center for the Advancement of Team Science, Analytics, and Systems Thinking in Health Services and Implementation Science Research, The Ohio State University, Columbus, OH USA; 2 Department of Biomedical Informatics, College of Medicine, The Ohio State University, Columbus, OH USA

## Past

A subset of patients with non-metastatic triple-negative breast cancer (TNBC) experience rapid relapse (rrTNBC), marked by aggressive clinical progression and high mortality within 24 months after diagnosis.^1^ Previous research has identified sociodemographic and treatment disparities associated with rrTNBC. Patients with rrTNBC have a higher likelihood of receiving Medicaid, having no insurance, being non-Hispanic black, or presenting at more advanced stages of disease. They also are less likely to receive both chemotherapy and surgery.^2,3^ Furthermore, extant research finds that patients with higher comorbidity are less likely to receive both treatments.^4^ Because higher comorbidity is highly correlated with age, this may drive treatment choices among older patients. As such, older patients with rrTNBC may be at risk of not receiving both treatments.

## Present

This study found that patients age 75 years or older, those with stage III cancer and those with multiple comorbidities were less likely to receive both treatments. Importantly, these patients (i.e., those who did not receive both treatments) had a triple to quadruple risk of rrTNBC compared with those who did receive both treatments. Thus, our study suggests that the combination of chemotherapy and breast surgery may be crucial in preventing rapid relapse.^5^ Additional findings from our study are consistent with previous literature suggesting that older age, public insurance, advanced TNBC stage, and higher comorbidity are linked to a higher risk of rapid relapse.^2,3^ These factors also were associated with a lower risk of receiving both chemotherapy and surgery.^5^

## Future

The current findings suggest that care providers should inform patients of all ages about the potential benefits of receiving both chemotherapy and surgery. Care providers may consider incorporating shared decision-making strategies to inform patients better regarding the benefits and risks of receiving both treatments versus not receiving them. Since this study defined rrTNBC as all-cause mortality at 24 months, future research is warranted in evaluating relapse itself as an outcome.
